# Evolution of a family of molecular Rube Goldberg contraptions

**DOI:** 10.1371/journal.pbio.3000405

**Published:** 2019-08-15

**Authors:** Morgan Beeby

**Affiliations:** Department of Life Sciences, Imperial College London, London, United Kingdom

## Abstract

Case studies of the evolution of molecular machines remain scarce. One of the most diverse and widespread homologous families of machines is the type IV filament (TFF) superfamily, comprised of type IV pili, type II secretion systems (T2SSs), archaella, and other less-well-characterized families. These families have functions including twitching motility, effector export, rotary propulsion, nutrient uptake, DNA uptake, and even electrical conductance, but it is unclear how such diversity evolved from a common ancestor. In this issue, Denise and colleagues take a significant step toward understanding evolution of the TFF superfamily by determining a global phylogeny and using it to infer an evolutionary pathway. Results reveal that the superfamily predates the divergence of Bacteria and Archaea, and show how duplications, acquisitions, and losses coincide with changes in function. Surprises include that tight adherence (Tad) pili were horizontally acquired from Archaea and that T2SSs were relatively recently repurposed from type IV pili. Results also enable better understanding of the function of the ATPase family that powers the superfamily. The study highlights the role of tinkering by exaptation—the repurposing of pre-existing functions for new roles—in the diversification of molecular machines.

## Abstract 2

Case studies of the evolution of molecular machines remain scarce. This Primer explores recent work describing the evolution of a functionally diverse family of such machines: the Bacterial and Archaeal Type IV filament family.

How life innovates has long fascinated humankind. Central to evolutionary innovation is exaptation, in which existing features are co-opted for new functions [1]. For example, avian flight evolved by exaptation of feathers for lift (feathers originated as insulators) [2], flexible wrists for wing folding (such wrists preceded flight) [3], and hollow bones for reduced weight (air pockets evolved with other functions and did not originally enter bones) [4], leading to an integrated flight system. In turn, such exaptations push the range of subsequent innovations into the “adjacent possible” [5]—in the case of flight, feathers were further exapted to become steering devices [6]. Thus, even with “nothing new under the sun,” evolution repurposes or combines previously disparate elements to create new functions.

Exaptation is also responsible for evolutionary innovations in the molecular machines of life. For example, injectisomes—nanoscale syringes used by Bacteria to pump toxins into our cells, causing diseases including food poisoning, chlamydia, and plague—have exapted the assembly process of bacterial flagella to secrete toxins [7,8]. Another particularly rich family of inter-related molecular machines is the type IV filament (TFF) superfamily, which function as grappling hooks for motility, lassos to capture DNA, straps to harness Bacteria together, propellers for swimming, pumps to export toxins, and siphons to import nutrients [9]. Such astonishing functional diversity in a homologous family of machines highlights the role of molecular exaptation, but how this diversity arose from a core machinery has been unclear, mainly due to the lack of an evolutionary framework to contextualize what is known.

The TFF superfamily is composed of a portfolio of molecular machines with diverse functions. First to be discovered were the namesake type IV pili, which extrude long, flexible filaments from the cell surface [9]. Type IV pili were historically subclassified based on differences in sequence lengths [10] into type IVa pili (T4aP), which can be sticky along their length for cell adhesion, retract as grappling hooks for jerky twitching motility [9], and bind DNA for uptake; less widespread type IVb pili (T4bP), associated with adhesion and microcolony formation during enteropathogenesis, including the toxin-coregulated pilus (Tcp) in *Vibrio cholerae* [11] and bundle-forming pilus in enteropathogenic *Escherichia coli* [12]; and, distinct from T4aP and T4bP, the tight adherence (Tad) pili, for cell adhesion [13]. More exotic roles include electrical conduction [14] and phage reception [15] by T4aP. Subsequently, type II secretion systems (T2SSs) were discovered to be TFF superfamily members. T2SSs push folded proteins through an outer membrane portal using a short dynamic pseudopilus related to other TFF pili (the distinction is functional: overexpression of pseudopilus components results in hyperpseudopili that resemble other TFF pili [16]). T2SS substrates can be enzymes or pathogenesis-associated effectors. Other curious TFF variants include archaella, which rotate helical pili as propellers despite being unrelated to bacterial flagella or eukaryotic cilia [17]; archaeal bindosomes for sugar uptake that may make pseudopili [18]; competence (Com) pili in monoderm Bacteria that bind DNA and retract for DNA uptake [9]; archaeal UV-induced pili (Ups) for DNA exchange and repair [9]; archaeal EppA dependent (Epd) pili [19]; and mannose-sensitive hemagglutinin (MSH) pili for surface attachment by pathogens.

Perhaps unsurprising for such a diverse family, nomenclature may confuse newcomers. Type IV pili are not to be confused with other unrelated, convergently evolved pili [20], or similarly named type IV secretion systems (T4SSs) that transfer DNA and deliver effectors into eukaryotic cells—particularly confusing given that the T4SS has a pilus called the T4SS-pilus. Similarly, T2SSs are not to be confused with type II pili. TFF pilins and pseudopilins contain a Class III signal peptide, not to be confused with the type III secretions systems at the core of the export apparatuses of injectisomes and flagella—rather, Class III signal peptides distinguish the N-terminal signal peptide in pilins from Class I signal peptides, which direct proteins for general secretion pathway (Sec) transport, and Class II signal peptides, which are lipoprotein sorting sequences. Furthermore, archaellum genes have recently been renamed from *fla* to *arl* [21] (here, I append the old names in parentheses), and homologs between different TFF families do not necessarily have corresponding naming systems; indeed, even homologs from the same family may have different names in different organisms.

Despite their functional differences, all TFFs have a conserved four-protein core that extrudes pili (or pseudopili) from the cell surface, supplemented by additional, lineage-specific proteins. The pilus is made of proteins called pilins, whose N-terminal Class III signal peptide is composed of a short hydrophilic sequence, ending with a conserved glycine and a 21-amino acid hydrophobic helix [9,19]. Major pilins are named PilA or PilE in T4aP, GspG in T2SSs, and ArlB (FlaB) in archaella. Pilins are translated into the membrane, where the second conserved component, a prepilin peptidase, cleaves the signal peptide after the glycine. This leaves the pilin as a transmembrane protein with an extracellular domain but no cytoplasmic residues [22]. The third conserved TFF component is a membrane platform protein made of three transmembrane domains that incorporates or removes pilins at the base of the pilus. Different techniques have all suggested that this protein dimerizes [23–26] and is called PilC or PilG in T4aP, GspF in T2SS [27], and ArlJ (FlaJ) in archaella. The fourth conserved component is a family of paralogous ATPases from the AAA+ ATPase superfamily that bind the cytoplasmic face of the membrane platform. Three variants are found: a universal “extension” ATPase that powers insertion of pilins from the membrane into extending pili (PilB or PilF in T4aP, GspE in T2SSs, ArlI [FlaI] in archaella) and two ATPases (PilT and less widespread PilU) that power removal of pilins from retracting pili, returning them to the membrane pool. Different conformations in the extension and retraction ATPases from T4aP suggest that the extension ATPase pushes against the membrane platform to incorporate pilins, while the retraction ATPase conversely pulls pilins out of the pilus [28]. The functions of these ATPases may, however, be more complex than first thought, as discussed below.

How, though, did the TFF superfamily evolve? This week in *PLOS Biology*, Denise, Abby, and Rocha [29] describe a global study of the phylogeny of the TFF superfamily to understand just this. Their study builds on earlier work that analyzed type IV pili and T2SSs [30], reviewed and surveyed homologies across species [9], and determined a phylogeny of archaeal TFF superfamily members [31]. The authors employed an elegant semiautomated approach to identify TFF systems that handles the complexity of duplicated, absent, substituted, or distantly diverged components using their previously developed Macromolecular System Finder, or MacSyFinder [32]. MacSyFinder facilitates context-based insights not possible from sequence alone, building on previous studies on other microbial systems [29,33]. The approach identifies mandatory or accessory proteins with hidden Markov models, then applies rules based on patterns of occurrence and genomic co-localization to identify TFF families.

The authors used this approach to identify TFF protein components across the Bacteria. The authors first consolidated protein families by identifying difficult-to-detect interfamily relationships. Using their global data set, they found that the ATPase and membrane platform together produced robust phylogenies of the core system, while the major pilin and the prepilin peptidases were less useful. They then used the related non-TFF AAA+ ATPase FtsK to determine the root of the ATPase phylogeny. FtsK is particularly suitable as it is only ever present in a single copy and is never horizontally transferred. Finally, the authors produced a rooted phylogeny of the entire TFF superfamily by transferring the ATPase root to a phylogeny estimated from a concatenation of the ATPase and membrane platform protein sequences.

The results are a fascinating bird’s-eye view of the entire TFF superfamily, enabling the authors to propose how its diversity evolved (Fig 1). One conclusion is immediately clear: the superfamily splits into two broad clades representing Bacteria and Archaea, indicating that TFF are ancient and were present in the last universal common ancestor (LUCA). This suggests that the progenitor TFF superfamily member function was to take up DNA, consistent with horizontal gene transfer being commonplace at the dawn of cellular life [34].

**Fig 1:**
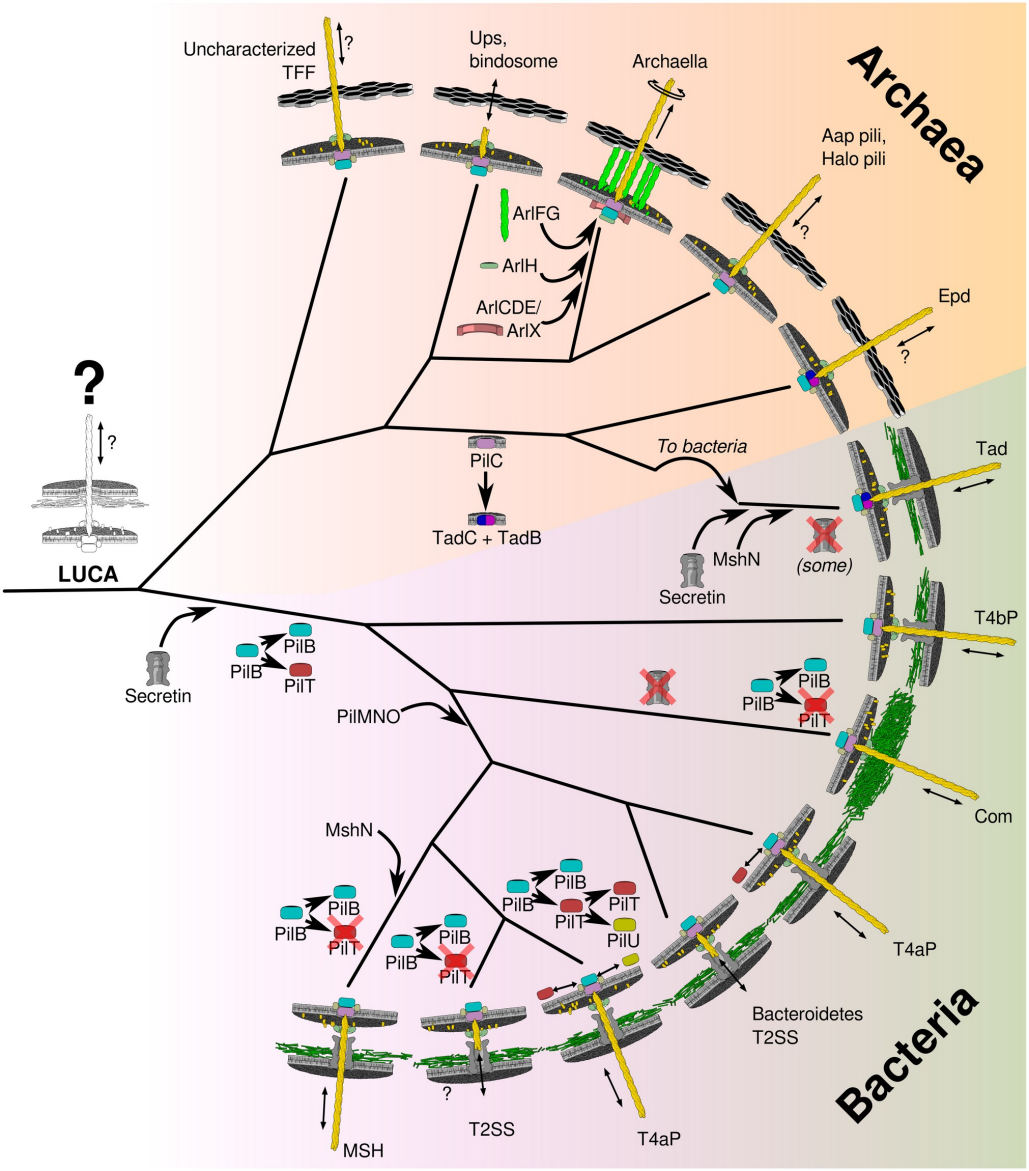
The global phylogeny of the TFF superfamily suggests their evolutionary pathway. A schematic based on the results from Denise and colleagues [29] capturing salient branch topologies and selected protein acquisition and loss events anticipated by parsimony. The upper major clade represents archaeal TFF superfamily members; the lower major clade represents Bacteria. The phylogeny suggests that the TFF superfamily was present in the LUCA. Archaea and Bacteria evolved distinct TFF families based on nuanced changes in core and accessory components, and Tad pili were a horizontal transfer from Archaea to Bacteria. Black hexagons represent archaeal S-layer; green mesh represents bacterial peptidoglycan. Aap, archaeal adhesive pilus; Com, competence; Epd, EppA dependent; LUCA, last universal common ancestor; MSH, mannose-sensitive hemagglutinin; Tad, tight adherence; TFF, type IV filament; T2SS, type II secretion system; T4aP, type IVa pilus; T4bP, type IVb pilus; Ups, UV-inducible pilus.

The archaeal TFF branch agrees with a recent study by Makarova and colleagues [31], although it unearths a surprising new discovery (Fig 1). After small basal clades of unknown function, the archaeal branch divides into two major clades: one featuring rotary archaella, adhesive *Halobacteria* pili, archaeal adhesive pilus (Aap) pili, sugar-uptake bindosomes, UV-inducible Ups pili, and as-yet-uncharacterized TFF systems; and a second clade clustering Epd pili [19] with—unexpectedly—bacterial Tad pili. This unanticipated finding is supported by congruency of phylogenies of individual components; shared fission of their membrane platform protein into two fragments; the same accessory components; short pilins; and similar genetic organization, making it clear that Tad pili, until now thought to be bacterial, were horizontally transferred from the Archaea. The transfer of a proto-Tad system was likely to a diderm Bacterium, as it coincided with recruitment of a secretin as a portal through the outer membrane; secretins are not required by Archaea because they lack an outer membrane. The secretin came from a pre-existing bacterial T4aP, suggesting that most bacterial TFF families were already present at the time of this transfer. It is satisfying to find that Tad pili are found almost exclusively in a single locus, facilitating their horizontal transfer as a single unit multiple times, explaining their widespread occurrence across Bacteria.

The bacterial TFF branch shows a ladder of branching TFF families (Fig 1). Unlike the archaeal branch, the ancestral Bacterial ATPase duplicated to produce a retraction ATPase at the root of the bacterial tree, indicating that the original bacterial TFFs were retractile. A secretin was also acquired at this stage, consistent with a diderm last bacterial common ancestor [35]. The T4bP branched first, confirming that T4aP and T4bP are genuinely distinct families and that the “type IV pili” grouping is paraphyletic. The monoderm-associated Com pili diverged next, with corresponding loss of their secretin. This left the T4aP, from which diverged the MSH pili and the T2SSs. Intriguingly, a so-called T2SS from *Cytophaga* branched from the T4aP and not from other T2SSs. Furthermore, the so-called T2SS from *Chlamydia* is chimaeric. These results distinguish these two “T2SSs” from true T2SSs.

These unexpected results suggest that secretion systems evolved within the TFF superfamily multiple times, and are relatively modern innovations. Naively, it has been thought that type IV pili evolved from “simpler” T2SSs. Instead, T2SSs branch relatively recently from the T4aP. Indeed, that the *Cytophaga* and *Chlamydia* “T2SSs” are not bona fide T2SSs suggests that T4aP were exapted to form T2SS-esque systems multiple times. Whether there is something particularly suitable about T4aP for such exaptation is unclear, and discovery of other independent T2SS-esque systems in the future will help better understand this. The finding echoes the independent emergence of secretion systems from bacterial flagella at least five times, with injectisomes, a secretion system in *Buchnera aphidicola*, and secreting flagella in *Bacillus*, *Campylobacter*, and *Yersina*. Indeed, it was also first thought that flagella evolved from “simpler” injectisomes, when the opposite is actually true. That T2SSs and injectisomes evolved relatively recently from T4aP and flagella, respectively, may be due to host-associated lifestyles emerging only relatively recently in Bacteria as Eukaryotes became potential niches.

One of the most significant contributions of the study is that it helps make sense of the function of the ATPases. Complements of ATPases vary in different bacterial TFF families. Although it was thought that adhesive pili lack retraction ATPases because they have no need to retract, sufficient evidence has accumulated to take seriously the idea that “extension” ATPases can also drive retraction in these pili. Denise and colleagues’ study makes it clear that these reports are distributed across the breadth of the TFF superfamily, implying that both extension and retraction by a single ATPase was an ancestral trait: Tad pili lacking a retraction ATPase can retract powered by their sole ATPase, albeit with lower force—approximately one sixth that exerted by T4aP [9,36,37]; T4bP are involved in twitching motility despite lacking a retraction ATPase [38]; Ups and Com pili facilitate DNA transfer and retract despite lacking a retraction ATPase [9,39]; and T4aP retract even after deletion of their retraction ATPase, albeit with lower force and speed [40]. This suggests that a bifunctional ancestral ATPase duplicated to form two ATPases, which sub-functionalized into dedicated extension and retraction ATPases. Intriguingly, TFF ATPases may form heterohexamers [41], which would eliminate the need to swap extension and retraction homohexamers. Sub-functionalization would then enable dedicated ATPases to optimize their individual functions. Duplication of an ancestral bifunctional ATPase protomer would by default lead to assembly of heterohexamers until mutations abolished heteromer binding interfaces. This could also explain how the secondary retraction ATPase, PilU, evolved. PilU has been implicated in producing higher retraction forces [42,43], which may correspond to the high force of T4aP retraction. Indeed, PilU cannot retract without PilT, further supporting that mixed-ability heterohexamers form [44]. Nevertheless, because the ATPases are capable of both extension and retraction, loss of the dedicated retraction ATPase could be mitigated by regain of the retraction function by the extension ATPase, as seen in various branches of the TFF superfamily that have lost their retraction ATPases. What controls switching between extension or retraction activity in a heterohexamer remains to be established.

The field is now poised to probe how molecular exaptations were implemented. Denise and colleagues’ study necessarily focuses on core components, and it will be interesting to now focus on changes in noncore components and how they contributed to exaptations of core protein functions. For example, what alterations enabled the sole archaellar ATPase, ArlI (FlaI), to power both assembly and rotation of archaellar filaments? ArlI drives both 36° and 60° rotations of the archaellum [45], suggesting a link to its hexameric structure, which may have been exapted to drive rotation. Was this putative exaptation intrinsic to changes in ArlI, or did ArlI’s rotary function simply require extrinsic changes in the noncore components, such as recruitment of coupling proteins ArlH (FlaH), ArlX (FlaX), or ArlCDE (FlaCDE); or anchoring by proposed stator complex ArlF (FlaF) [46]? Similarly, how did T2SSs develop the ability to secrete effectors using an exapted pseudopilus [47]? More comparative genomics, more structures, and more biophysical studies will be needed to better understand such transitions.

The study also lays groundwork to understand how noncore components are recruited during evolution. What were the origins of newly recruited proteins and what, if anything, poised them for recruitment? Future studies on the origins of secretins and factors that make them predisposed to co-option may be particularly insightful here. Secretins are modular and easily horizontally acquired. Bacterial TFFs first recruited a secretin and later donated it to the incoming Tad system from the Archaea, injectisomes have recruited secretins multiple times, and secretins are also found in filamentous phages [48].

Another ambitious future goal will be to understand the origins of the TFFs. The only core component with clear non-TFF homologs is the AAA+ ATPase family. The AAA+ ATPases are an incredibly diverse family [49] that tend to form hexamers that undergo cyclical conformation changes with ATP hydrolyses. These conformational changes hint that a rotary function was always present for exaptation for extension, retraction, or rotation. Whether speculative homologies of the membrane platform protein to ABC transporters or ATP synthase components are correct will require structural verification [29].

Denise and colleagues highlight that at the heart of evolution of the TFF superfamily has been evolutionary tinkering using the tools at hand: no radically new mechanisms or components have been introduced to enable the functional diversity of the TFF. Rather, pre-existing functions of pre-existing components have been exapted to shift the function of existing machinery. Perhaps unsurprisingly, evolution the tinkerer has built Rube Goldberg (or, east of the Atlantic, Heath Robinson) contraptions [50]: the piston-pumping T2SS, the whirling archaellum, the sugar-siphoning bindosome, and other devices, using only the material at hand—an ancestral, DNA-lassoing type IV pilus.

## Abbreviations

Com: competence
Epd: EppA dependent
LUCA: last universal common ancestor
MSH: mannose-sensitive hemagglutinin
Sec: general secretion pathway
T2SS: type II secretion system
T4aP: type IVa pilus
T4bP: type IVb pilus
T4SS: type IV secretion system
Tad: tight adherence
Tcp: toxin-coregulated pilus
TFF: type IV filament
UPS: UV-inducible pili

## References

[1] Gould SJ, Vrba ES. Exaptation-A Missing Term in the Science of Form. Paleobiology. 1982;8: 4–15.

[2] Dial KP. Wing-assisted incline running and the evolution of flight. Science. 2003;299: 402–404. 10.1126/science.1078237 12532020

[3] Corwin Sullivan, Hone David W. E., Xu Xing, Zhang Fucheng. The asymmetry of the carpal joint and the evolution of wing folding in maniraptoran theropod dinosaurs. Proceedings of the Royal Society B: Biological Sciences. 2010;277: 2027–2033. 10.1098/rspb.2009.2281 20200032 PMC2880093

[4] Wedel MJ. Origin of postcranial skeletal pneumaticity in dinosaurs. Integrative Zoology. 2006;1: 80–85. 10.1111/j.1749-4877.2006.00019.x 21395998

[5] Kauffman SA. Investigations. Oxford, New York: Oxford University Press; 2003.

[6] Matyjasiak Piotr, Matyjasiak Jolanta, de Lope Florentino Møller Anders P. Vane emargination of outer tail feathers improves flight manoeuvrability in streamerless hirundines, Hirundinidae. Proceedings of the Royal Society of London Series B: Biological Sciences. 2004;271: 1831–1838. 10.1098/rspb.2004.2812 15315899 PMC1691798

[7] Abby SS, Rocha EPC. The non-flagellar type III secretion system evolved from the bacterial flagellum and diversified into host-cell adapted systems. PLoS Genet. 2012;8: e1002983 10.1371/journal.pgen.1002983 23028376 PMC3459982

[8] Rossmann FM, Beeby M. Insights into the evolution of bacterial flagellar motors from high-throughput in situ electron cryotomography and subtomogram averaging. Acta Cryst D, Acta Cryst Sect D, Acta Crystallogr D, Acta Crystallogr Sect D, Acta Crystallogr D Struct Biol, Acta Crystallogr Sect D Struct Biol. 2018;74 10.1107/S2059798318007945 29872008 PMC6096493

[9] Berry J-L, Pelicic V. Exceptionally widespread nanomachines composed of type IV pilins: the prokaryotic Swiss Army knives. FEMS Microbiol Rev. 2015;39: 134–154. 10.1093/femsre/fuu001 25793961 PMC4471445

[10] Giltner CL, Nguyen Y, Burrows LL. Type IV Pilin Proteins: Versatile Molecular Modules. Microbiology and Molecular Biology Reviews. 2012;76: 740–772. 10.1128/MMBR.00035-12 23204365 PMC3510520

[11] Boyd EF, Waldor MK. Evolutionary and functional analyses of variants of the toxin-coregulated pilus protein TcpA from toxigenic Vibrio cholerae non-O1/non-O139 serogroup isolates. Microbiology. 2002;148: 1655–1666. 10.1099/00221287-148-6-1655 12055286

[12] Cleary J, Lai L-C, Shaw RK, Straatman-Iwanowska A, Donnenberg MS, Frankel G, et al Enteropathogenic Escherichia coli (EPEC) adhesion to intestinal epithelial cells: role of bundle-forming pili (BFP), EspA filaments and intimin. Microbiology. 2004;150: 527–538. 10.1099/mic.0.26740-0 14993302

[13] Sangermani M, Hug I, Sauter N, Pfohl T, Jenal U. Tad Pili Play a Dynamic Role in Caulobacter crescentus Surface Colonization. mBio. 2019;10: e01237–19. 10.1128/mBio.01237-19 31213565 PMC6581867

[14] Reardon PN, Mueller KT. Structure of the Type IVa Major Pilin from the Electrically Conductive Bacterial Nanowires of Geobacter sulfurreducens. J Biol Chem. 2013; jbc.M113.498527. 10.1074/jbc.M113.498527 23965997 PMC3795228

[15] Whitchurch CB, Hobbs M, Livingston SP, Krishnapillai V, Mattick JS. Characterisation of a Pseudomonas aeruginosa twitching motility gene and evidence for a specialised protein export system widespread in eubacteria. Gene. 1991;101: 33–44. 10.1016/0378-1119(91)90221-v 1676385

[16] Durand É, Bernadac A, Ball G, Lazdunski A, Sturgis JN, Filloux A. Type II Protein Secretion in Pseudomonas aeruginosa: the Pseudopilus Is a Multifibrillar and Adhesive Structure. Journal of Bacteriology. 2003;185: 2749–2758. 10.1128/JB.185.9.2749-2758.2003 12700254 PMC154417

[17] Albers S-V, Jarrell KF. The Archaellum: An Update on the Unique Archaeal Motility Structure. Trends in Microbiology. 2018;26: 351–362. 10.1016/j.tim.2018.01.004 29452953

[18] Zolghadr B, Klingl A, Rachel R, Driessen AJM, Albers SV. The bindosome is a structural component of the Sulfolobus solfataricus cell envelope. Extremophiles. 2011;15: 235–244. 10.1007/s00792-010-0353-0 21234771 PMC3047682

[19] Szabó Z, Stahl AO, Albers S-V, Kissinger JC, Driessen AJM, Pohlschröder M. Identification of Diverse Archaeal Proteins with Class III Signal Peptides Cleaved by Distinct Archaeal Prepilin Peptidases. J Bacteriol. 2007;189: 772–778. 10.1128/JB.01547-06 17114255 PMC1797317

[20] Ottow JCG. Ecology, Physiology, and Genetics of Fimbriae and Pili. Annual Review of Microbiology. 1975;29: 79–108. 10.1146/annurev.mi.29.100175.000455 1180526

[21] Pohlschroder M, Pfeiffer F, Schulze S, Halim MFA. Archaeal cell surface biogenesis. FEMS Microbiol Rev. 2018;42: 694–717. 10.1093/femsre/fuy027 29912330 PMC6098224

[22] Dupuy B, Taha MK, Pugsley AP, Marchal C. Neisseria gonorrhoeae prepilin export studied in Escherichia coli. Journal of Bacteriology. 1991;173: 7589–7598. 10.1128/jb.173.23.7589-7598.1991 1938955 PMC212527

[23] Chang Y-W, Rettberg LA, Treuner-Lange A, Iwasa J, Søgaard-Andersen L, Jensen GJ. Architecture of the type IVa pilus machine. Science. 2016;351: aad2001 10.1126/science.aad2001 26965631 PMC5929464

[24] Iwata S, Kinosita Y, Uchida N, Nakane D, Nishizaka T. Motor torque measurement of Halobacterium salinarum archaellar suggests a general model for ATP-driven rotary motors. Communications Biology. 2019;2: 199 10.1038/s42003-019-0422-6 31149643 PMC6534597

[25] Karuppiah V, Hassan D, Saleem M, Derrick JP. Structure and oligomerization of the PilC type IV pilus biogenesis protein from Thermus thermophilus. Proteins: Structure, Function, and Bioinformatics. 2010;78: 2049–2057. 10.1002/prot.22720 20455262

[26] Takhar HK, Kemp K, Kim M, Howell PL, Burrows LL. The Platform Protein Is Essential for Type IV Pilus Biogenesis. J Biol Chem. 2013;288: 9721–9728. 10.1074/jbc.M113.453506 23413032 PMC3617274

[27] Korotkov KV, Sandkvist M, Hol WGJ. The type II secretion system: biogenesis, molecular architecture and mechanism. Nature Reviews Microbiology. 2012;10: 336–351. 10.1038/nrmicro2762 22466878 PMC3705712

[28] McCallum M, Tammam S, Khan A, Burrows LL, Howell PL. The molecular mechanism of the type IVa pilus motors. Nature Communications. 2017;8: ncomms15091 10.1038/ncomms15091 28474682 PMC5424180

[29] Denise R, Abby SS, Rocha EPC. Diversification of the type IV filament super-family into machines for adhesion, protein secretion, DNA uptake and motility. PLoS Biol. 2019;17(7): e3000390 10.1371/journal.pbio.3000390 31323028 PMC6668835

[30] Peabody CR, Chung YJ, Yen M-R, Vidal-Ingigliardi D, Pugsley AP, Saier MH. Type II protein secretion and its relationship to bacterial type IV pili and archaeal flagella. Microbiology (Reading, England). 2003;149: 3051–72. 10.1099/mic.0.26364-0 14600218

[31] Makarova KS, Koonin EV, Albers SV. Diversity and evolution of type IV pili systems in Archaea. Frontiers in Microbiology. 2016;7 10.3389/fmicb.2016.00667 27199977 PMC4858521

[32] Abby SS, Néron B, Ménager H, Touchon M, Rocha EPC. MacSyFinder: A Program to Mine Genomes for Molecular Systems with an Application to CRISPR-Cas Systems. PLoS ONE. 2014;9: e110726 10.1371/journal.pone.0110726 25330359 PMC4201578

[33] Abby SS, Cury J, Guglielmini J, Néron B, Touchon M, Rocha EPC. Identification of protein secretion systems in bacterial genomes. Scientific Reports. 2016;6: 23080 10.1038/srep23080 26979785 PMC4793230

[34] Woese CR. On the evolution of cells. Proc Natl Acad Sci USA. 2002;99: 8742–8747. 10.1073/pnas.132266999 12077305 PMC124369

[35] Antunes LC, Poppleton D, Klingl A, Criscuolo A, Dupuy B, Brochier-Armanet C, et al Phylogenomic analysis supports the ancestral presence of LPS-outer membranes in the Firmicutes. Eisen J, editor. eLife. 2016;5: e14589 10.7554/eLife.14589 27580370 PMC5007114

[36] Ellison CK, Kan J, Dillard RS, Kysela DT, Ducret A, Berne C, et al Obstruction of pilus retraction stimulates bacterial surface sensing. Science. 2017;358: 535–538. 10.1126/science.aan5706 29074778 PMC5805138

[37] Ellison CK, Kan J, Chlebek JL, Hummels KR, Panis G, Viollier PH, et al A bifunctional ATPase drives tad pilus extension and retraction. bioRxiv. 2019; 616128 10.1101/616128 PMC6920026 31897429

[38] Mazariego-Espinosa K, Cruz A, Ledesma MA, Ochoa SA, Xicohtencatl-Cortes J. Longus, a Type IV Pilus of Enterotoxigenic Escherichia coli, Is Involved in Adherence to Intestinal Epithelial Cells. Journal of Bacteriology. 2010;192: 2791–2800. 10.1128/JB.01595-09 20348256 PMC2876479

[39] Laurenceau R, Péhau-Arnaudet G, Baconnais S, Gault J, Malosse C, Dujeancourt A, et al A Type IV Pilus Mediates DNA Binding during Natural Transformation in Streptococcus pneumoniae. PLoS Pathog. 2013;9: e1003473 10.1371/journal.ppat.1003473 23825953 PMC3694846

[40] Ellison CK, Dalia TN, Ceballos AV, Wang JC-Y, Biais N, Brun YV, et al Retraction of DNA-bound type IV competence pili initiates DNA uptake during natural transformation in Vibrio cholerae. Nature Microbiology. 2018;3: 773 10.1038/s41564-018-0174-y 29891864 PMC6582970

[41] Georgiadou M, Castagnini M, Karimova G, Ladant D, Pelicic V. Large-scale study of the interactions between proteins involved in type IV pilus biology in Neisseria meningitidis: characterization of a subcomplex involved in pilus assembly. Molecular Microbiology. 2012;84: 857–873. 10.1111/j.1365-2958.2012.08062.x 22486968

[42] Chlebek JL, Hughes HQ, Ratkiewicz AS, Rayyan R, Wang JC-Y, Herrin BE, et al PilT and PilU are homohexameric ATPases that coordinate to retract type IVa pili. bioRxiv. 2019; 634048 10.1101/634048 PMC6821130 31626631

[43] Talà L, Fineberg A, Kukura P, Persat A. Pseudomonas aeruginosa orchestrates twitching motility by sequential control of type IV pili movements. Nature Microbiology. 2019;4: 774 10.1038/s41564-019-0378-9 30804544 PMC6522360

[44] Brown DR, Helaine S, Carbonnelle E, Pelicic V. Systematic Functional Analysis Reveals That a Set of Seven Genes Is Involved in Fine-Tuning of the Multiple Functions Mediated by Type IV Pili in Neisseria meningitidis. Infection and Immunity. 2010;78: 3053–3063. 10.1128/IAI.00099-10 20439474 PMC2897404

[45] Kinosita Y, Uchida N, Nakane D, Nishizaka T. Direct observation of rotation and steps of the archaellum in the swimming halophilic archaeon Halobacterium salinarum. Nature Microbiology. 2016;1: 16148–16148. 10.1038/nmicrobiol.2016.148 27564999

[46] Banerjee A, Tsai C-L, Chaudhury P, Tripp P, Arvai AS, Ishida JP, et al FlaF Is a β-Sandwich Protein that Anchors the Archaellum in the Archaeal Cell Envelope by Binding the S-Layer Protein. Structure. 2015;23: 863–872. 10.1016/j.str.2015.03.001 25865246 PMC4425475

[47] Michel-Souzy S, Douzi B, Cadoret F, Raynaud C, Quinton L, Ball G, et al Direct interactions between the secreted effector and the T2SS components GspL and GspM reveal a new effector-sensing step during type 2 secretion. J Biol Chem. 2018;293: 19441–19450. 10.1074/jbc.RA117.001127 30337370 PMC6302157

[48] Korotkov KV, Gonen T, Hol WGJ. Secretins: dynamic channels for protein transport across membranes. Trends in Biochemical Sciences. 2011;36: 433–443. 10.1016/j.tibs.2011.04.002 21565514 PMC3155655

[49] Snider J, Houry WA. AAA+ proteins: diversity in function, similarity in structure. Biochemical Society Transactions. 2008;36: 72 10.1042/BST0360072 18208389

[50] Graur D. Molecular and Genome Evolution. Oxford, New York: Oxford University Press; 2016.

